# Postoperative delirium prediction using machine learning models and preoperative electronic health record data

**DOI:** 10.1186/s12871-021-01543-y

**Published:** 2022-01-03

**Authors:** Andrew Bishara, Catherine Chiu, Elizabeth L. Whitlock, Vanja C. Douglas, Sei Lee, Atul J. Butte, Jacqueline M. Leung, Anne L. Donovan

**Affiliations:** 1 Department of Anesthesia and Perioperative Care, University of California, San Francisco, 521 Parnassus Avenue, San Francisco, CA 94143 USA; 2 Bakar Computational Health Sciences Institute, University of California San Francisco, 490 Illinois Street, San Francisco, CA 94143 USA; 3 Weill Institute for Neurosciences and Department of Neurology, University of California, 505 Parnassus Avenue, San Francisco, CA 94143 USA; 4 Division of Geriatrics, University of California, San Francisco, 505 Parnassus Avenue, San Francisco, CA 94143 USA

**Keywords:** Postoperative delirium, Delirium prevention, Risk prediction model, Machine learning, Geriatric surgery

## Abstract

**Background:**

Accurate, pragmatic risk stratification for postoperative delirium (POD) is necessary to target preventative resources toward high-risk patients. Machine learning (ML) offers a novel approach to leveraging electronic health record (EHR) data for POD prediction. We sought to develop and internally validate a ML-derived POD risk prediction model using preoperative risk features, and to compare its performance to models developed with traditional logistic regression.

**Methods:**

This was a retrospective analysis of preoperative EHR data from 24,885 adults undergoing a procedure requiring anesthesia care, recovering in the main post-anesthesia care unit, and staying in the hospital at least overnight between December 2016 and December 2019 at either of two hospitals in a tertiary care health system. One hundred fifteen preoperative risk features including demographics, comorbidities, nursing assessments, surgery type, and other preoperative EHR data were used to predict postoperative delirium (POD), defined as any instance of Nursing Delirium Screening Scale ≥2 or positive Confusion Assessment Method for the Intensive Care Unit within the first 7 postoperative days. Two ML models (Neural Network and XGBoost), two traditional logistic regression models (“clinician-guided” and “ML hybrid”), and a previously described delirium risk stratification tool (AWOL-S) were evaluated using the area under the receiver operating characteristic curve (AUC-ROC), sensitivity, specificity, positive likelihood ratio, and positive predictive value. Model calibration was assessed with a calibration curve. Patients with no POD assessments charted or at least 20% of input variables missing were excluded.

**Results:**

POD incidence was 5.3%. The AUC-ROC for Neural Net was 0.841 [95% CI 0. 816–0.863] and for XGBoost was 0.851 [95% CI 0.827–0.874], which was significantly better than the clinician-guided (AUC-ROC 0.763 [0.734–0.793], *p* < 0.001) and ML hybrid (AUC-ROC 0.824 [0.800–0.849], *p* < 0.001) regression models and AWOL-S (AUC-ROC 0.762 [95% CI 0.713–0.812], *p* < 0.001). Neural Net, XGBoost, and ML hybrid models demonstrated excellent calibration, while calibration of the clinician-guided and AWOL-S models was moderate; they tended to overestimate delirium risk in those already at highest risk.

**Conclusion:**

Using pragmatically collected EHR data, two ML models predicted POD in a broad perioperative population with high discrimination. Optimal application of the models would provide automated, real-time delirium risk stratification to improve perioperative management of surgical patients at risk for POD.

**Supplementary Information:**

The online version contains supplementary material available at 10.1186/s12871-021-01543-y.

## Introduction

Postoperative delirium (POD) is a common and serious complication of surgery [1], and is associated with numerous adverse events including prolonged length of stay, more frequent institutional discharge, higher readmission rates, functional decline, dependency in activities of daily living, and cognitive decline [2–9]. Many cases of POD can be prevented with multicomponent non-pharmacologic interventions [10], the Hospital Elder Life Program [11], or perioperative geriatric consultations [12, 13]. Effective perioperative interventions combining delirium risk stratification with focused delirium prevention care practices have been described [14], though further improvement in the discrimination of the delirium risk stratification tool used in this intervention [15] could allow for better targeting of finite resources to the patients who need them most. Since preventative interventions require significant time and energy from busy clinicians [16], improving and automating risk stratification procedures is critically important.

Machine learning-derived risk prediction models have been developed to predict delirium in hospitalized patients [17], postoperative delirium in focused patient populations [18], non-delirium-related intraoperative complications [19, 20], and postoperative mortality [21], in addition to applications in many other contexts [22]. Machine learning (ML) methodology has the potential to improve upon existing POD risk prediction models in many important ways. Whereas existing delirium prediction models tend to rely on well-known delirium risk factors such as age and cognitive impairment [23–25], ML allows for analysis of patterns in large amounts of data pragmatically collected in the electronic health record (EHR) to identify higher-order interactions that would be difficult to identify through traditional data analysis techniques [26]. In addition, increasingly feasible real-time EHR-based applications of ML-derived predictions in clinical practice [22, 26] have the potential to conserve valuable human resources through automation of risk stratification procedures, since use of existing risk stratification tools have often required too much clinician input to enter clinical workflow [24].

We sought to develop and internally validate a ML-derived model for the automated prediction of postoperative delirium in a broad surgical patient population using only pragmatically collected EHR-based data elements available prior to the start of surgery. We compare the performance of two ML models (gradient boosting and artificial neural network) against both traditional logistic regression and the POD risk stratification tool currently used at our institution, hypothesizing that use of ML to model POD risk would outperform other methods.

## Methods

This manuscript was prepared in accordance with the TRIPOD guidelines [27]. Approval for a retrospective review of the EHR was obtained by the University of California, San Francisco Institutional Review Board (IRB #18-26,109), and the requirement for written informed consent was waived. The study was conducted in accordance with all requirements outlined by the IRB.

### Study population

This study included all encounters in patients ages 18 and over undergoing surgery or a procedure requiring anesthesia care and staying in the hospital at least overnight at either of two adult hospitals in a non-trauma tertiary care health system between December 2016 and December 2019. All adults were included to allow for application of the model broadly in the perioperative setting. Moffitt-Long Hospital is the health system’s largest hospital, housing a wide variety of surgical and procedural specialties including high volume neurosurgery and transplant surgery services. Surgical services at Mission Bay Hospital include various surgical subspecialties, primarily focusing on cancer surgery. Procedures requiring anesthesia recovery outside of the main post-anesthesia care unit were excluded, as were patients who were discharged on the same day as their procedure. Patients who had no POD assessments charted and patients missing data for at least 20% of input variables were also excluded.

### Measures

#### Predictors

A total of 115 predictor variables derived solely from preoperative characteristics were given as input to each model (Additional file 1, Table S1). Variables included were selected by the authors from those used in a ML-derived model developed to predict incident delirium in hospitalized medical patients [17]. Variables selected were relevant to surgical patients and consistently available in the EHR prior to surgery. Only preoperative variables were included to allow delirium prediction to occur at the start of surgery, so that anesthesia providers and surgeons would be able to immediately adjust the intraoperative and postoperative management strategy. AWOL-S is an EHR-based risk stratification tool in which predefined locally-derived regression coefficients are applied to each of five terms [Age, ability to spell WORLD backward, Orientation to place, American Society of Anesthesiologists Classification (iLlness severity), and procedure-specific Surgical risk] to calculate a predicted risk of POD for an individual patient [15]. Each of these individual terms was included as a predictor variable for ML models.

#### Outcomes

The POD outcome was defined as any instance of Nursing Delirium Screening Scale (NuDESC) [28] score ≥ 2 or positive Confusion Assessment Method for the Intensive Care Unit (CAM-ICU) [29] recorded during the first seven postoperative days. Patients were assessed by their bedside nurse at least once every 12-h shift using NuDESC on acute care wards or CAM-ICU in the ICU. Bedside nurses were trained in delirium assessment as part of a hospital-wide delirium care program [30].

### Data collection and preprocessing

All patient data were gathered from the EHR (Epic, Verona, WI) using a unique surgical encounter code. Missing numeric values were rare and were therefore substituted with the population mean [31], and missing categorical values were labeled as ‘unknown.’ The entire dataset was randomly split into a training dataset (80%) and a test dataset (20%) for model development. All numeric variables in the training dataset were rescaled such that absolute values were contained between 0 and 1, so that numeric variables with higher absolute values would not be weighed inappropriately higher than those with lower absolute values. All categorical variables were converted into indicator variables with values of 0 (no/absent) or 1 (yes/present). 20% of the training dataset (i.e., 16% of the overall dataset) was reserved for a validation dataset used to tune hyperparameters. The test dataset was left untouched throughout all model development. After each respective model was fully developed, the numeric and categorical variables in the test dataset were normalized based on the same rules determined by the training dataset. The end result was the following: training dataset (15,926 patients), validation dataset (3982 patients), and test dataset (4977 patients).

### Statistical analysis

Descriptive statistics and ML model development were performed using R [32] and Python, respectively. For numeric variables, differences in mean and standard deviation were tested with the t-test if parametric, and differences in median and interquartile range were tested with the Kruskal-Wallis test if non-parametric. For categorical variables, chi-square was used for comparisons, unless expected cell frequencies were less than 5, in which case Fisher’s exact test was used.

### Model development

Each machine learning model approaches classification problems in a unique way. Therefore, individual models have relative strengths and weaknesses in making predictions. Because POD pathophysiology is complex with incompletely understood interactions between risk factors, we selected two ML models to allow us to identify the model that most accurately predicts POD using our rich dataset.

#### Gradient boosting

We used the eXtreme Gradient Boosting (XGBoost) algorithm to train a decision tree-based model on the training dataset. XGBoost was chosen for its robustness to overfitting and interpretability of results. The validation dataset was used to fine-tune specific hyperparameters (learning rate = 0.01, maximum tree depth = 4, minimum child weight = 2, number of estimators = 1000, scaled positive weight = 1) with 10-fold cross-validation. Using the feature importance summary plot function of the SHapley Additive exPlanations (SHAP) package, we visualized the 20 most influential prediction variables chosen by XGBoost [33].

#### Neural network

We used TensorFlow [34] to build a neural network consisting of three sequential layers: a densely connected hidden layer, a dropout layer, and a sigmoid-based output layer. Neural network was chosen because of its ability to find complex interactions between variables. The neural network was trained on the training dataset in batch sizes of 1500 cases over 10 epochs. The validation dataset was used to determine proper weights to account for the unbalanced distribution of outcomes.

#### Multivariable logistic regression

Traditional logistic regression is one of the most widely used and accepted modeling methods in medicine, and performance of logistic regression has been competitive to that of machine learning in many settings [35, 36]. Using fewer risk features may allow for simpler, more interpretable, point-based models that are easier to implement into clinical practice. Thus, two multivariable logistic regression models were created for comparison to ML-derived models: one based on an existing POD prediction model (“clinician-guided”), and one based on variables selected by ML (“ML hybrid”). We based the clinician-guided regression model on a 20-variable model predicting POD in older patients using data from the multi-institution American College of Surgeons National Surgery Quality Improvement Project (ACS NSQIP) database [37]. Seventeen of the 20 variables were available in our EHR; three (work relative value units, wound class, and surrogate consent) were not. In some cases, a surrogate variable with the same general clinical implication was substituted for the exact variable reported by Berian, et al., when data for the exact variable did not exist in our EHR (e.g., inability to spell WORLD backward was used as a marker for preoperative cognitive impairment).

For the ML hybrid model, 18 out of the 20 predictor variables derived from the feature importance summary of an iteration of the XGBoost algorithm (using the following hyper-parameters in XGBoost model development: learning rate = 0.05, maximum tree depth = 7, minimum child weight = 7, number of estimators = 150, scaled positive weight = 10) were included. Four variables which had considerable overlap were combined into two variables without changing the clinical implication [i.e., inpatient (risk) and outpatient (protective) patient status, unable (risk) and able (protective) to spell ‘WORLD’ backwards].

#### Comparison to AWOL-S

The ML-derived models were also compared to AWOL-S, the POD risk stratification tool used in our institution. Risk stratification with AWOL-S is performed preoperatively for all adult surgical patients; a calculated probability of delirium of 5% or greater is considered “high risk.” For those terms not already in the EHR (i.e., WORLD backward and orientation to place), assessments are performed and documented by preoperative nurses. Sensitivity, specificity, positive likelihood ratio, and positive predictive value of AWOL-S were calculated at a predicted POD risk (i.e., threshold or cutoff value) of 5%.

### Model evaluation

Model performance was evaluated based on the area under the receiver operating characteristic curve (AUC-ROC), and confidence intervals (CI) were derived from 10-fold cross validation (CV) and DeLong’s method (DL) [38, 39]. For each ML model, an optimal decision threshold, defined as the threshold at which the sum of sensitivity and (1-specificity) is greatest, was determined on the validation dataset for subsequent calculation of model sensitivity, specificity, positive likelihood ratio, positive predictive value, and negative predictive value. Because AWOL-S was previously validated on a separate dataset, cross validation was not applicable, and performance was evaluated on AUC-ROC with confidence intervals derived by DeLong’s method. A calibration plot was generated for each model.

## Results

**Fig. 1:**
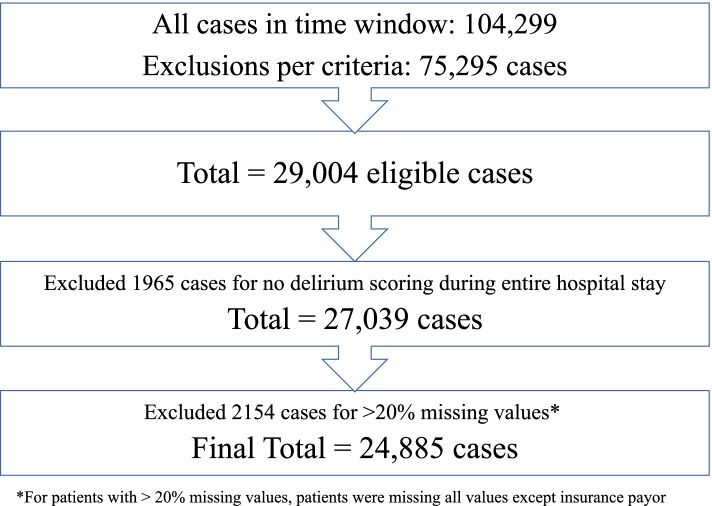
Inclusion flow diagram

Twenty-nine thousand four surgical encounters were evaluated. After exclusion of 4119 encounters (1965 patients with no delirium score ever recorded and 2154 patients with > 20% missing variables), 24,885 surgical encounters were included in the analysis (Fig. 1). 77,125/81,515 (94.6%) of total delirium assessments were performed using NuDESC, while the remainder were performed using CAM-ICU. The overall incidence of delirium was 5.3%. 325/4390 (7.4%) screens performed using CAM-ICU were positive, while 1373/77,125 (1.8%) screens performed using NuDesc were positive. Patients who developed delirium were older, more likely male, and had more comorbidities and a higher American Society of Anesthesiologists Classification (Table 1). Patients developing delirium were also more likely to have undergone inpatient surgery, emergency surgery, and/or neurological surgery.

**Table 1:** Participant Characteristics

***Variable***	***No Delirium*** *(n = 23,558)*	***Delirium*** *(n = 1327)*	***p-value***	***% Missing***
Age (mean (SD))	59.24 (15.29)	67.41 (15.02)	< 0.001	0
Sex (%)				0
Male	11,607 (49.3)	669 (50.4)	0.033	
ASA Class (%)				0.7
1	1196 (5.1)	6 (0.5)	< 0.001	
2	11,522 (49.2)	319 (24.3)	< 0.001	
3	9948 (42.5)	879 (67.0)	< 0.001	
4	728 (3.1)	105 (8.0)	< 0.001	
Emergent Case (%)	2220 (9.4)	353 (26.6)	< 0.001	0
Inpatient (%)	10,613 (45.1)	930 (70.1)	< 0.001	0
Surgical Service (%)^a^				0
Neurological Surgery	3391 (14.4)	420 (31.7)	< 0.001	
Orthopedics Surgery	5911 (25.1)	278 (20.9)	< 0.001	
General Surgery	4975 (21.1)	239 (18.0)	0.007	
Vascular Surgery	1081 (4.6)	127 (9.6)	< 0.001	
Genito-Urologic Surgery	2356 (10.0)	47 (3.5)	< 0.001	
Otolaryngology-Head and Neck Surgery	992 (4.2)	33 (2.5)	0.002	
Transplant Surgery	1122 (4.8)	26 (2.0)	< 0.001	
Gynecologic Oncology	648 (2.8)	22 (1.7)	0.02	
Thoracic Surgery	540 (2.3)	16 (1.2)	0.01	
Primary Language (%)^b^				0
English	21,513 (91.3)	1195 (90.1)	0.124	
Spanish	992 (4.2)	45 (3.4)	0.166	
Chinese - Cantonese	315 (1.3)	36 (2.7)	< 0.001	
Unable to spell WORLD backwards (%)	1367 (5.8)	259 (19.5)	< 0.001	0
Not oriented to place (%)	468 (2.0)	134 (10.1)	< 0.001	0
History of Diabetes (%)	4537 (19.3)	385 (29.0)	< 0.001	0
History of Chronic Kidney Disease (%)	2269 (9.6)	155 (11.7)	0.016	0
History of Heart Failure (%)	987 (4.2)	109 (8.2)	< 0.001	0
Smoking History (%)	6241 (26.5)	522 (39.3)	< 0.001	0

*Abbreviations: SD* standard deviation, *ASA* American Society of Anesthesiologists

^a^Nine surgical services with the highest patient volume (out of 19 total services) are listed

^b^Three language categories with the largest number of patients (out of 8 total categories) are listed

**Fig. 2:**
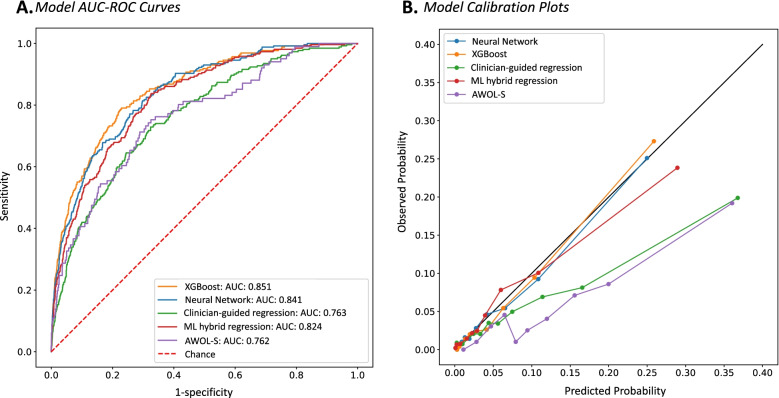
Model AUC-ROC curves and calibration plots. **A** Receiver Operating Characteristic curves for five POD prediction models. **B** Calibration plots for five POD prediction models. XGBoost (orange), Neural network (blue), Clinician-guided regression (green), ML hybrid regression (red), AWOL-S (purple)

**Fig. 3:**
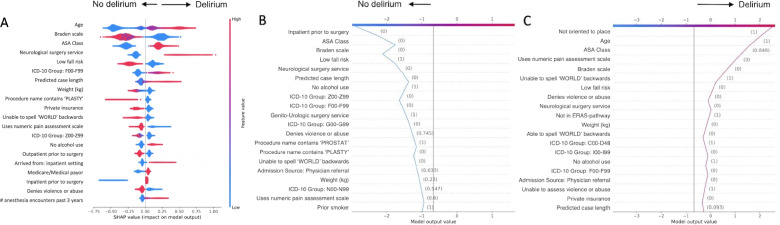
Visualization of decisions made by the XGBoost algorithm. **A** Top 20 most influential variables used by XGBoost. *Interpretation*: *Each dot represents a variable for an individual patient instance. Variables pictured to the right side of the y-axis influenced the model to predict delirium, whereas variables to the left of the y-axis influenced the model against prediction of delirium. Red signifies a higher absolute value (numeric variables) or yes/present (categorical variables), and blue signifies a lower absolute value (numeric variables) or no/absent (categorical variables). For example: Higher age (red color) influenced the model to predict delirium (right of y-axis), whereas lower age (blue color) influenced the model toward prediction of no delirium.* Decision path for a true negative (**B**) and a true positive (**C**) delirium prediction by XGBoost for two individual patients. *Interpretation: The algorithm begins at the center of the x-axis with a baseline value. The model considers each variable along the y-axis one at a time (values shown in parenthesis), to influence the model toward making a positive (vertical line moves toward the right) or negative (vertical line moves toward the left) delirium risk prediction. For example: In panel* ***B****, variables which significantly influenced the model toward a negative delirium prediction include outpatient surgery, ASA class 1, low fall risk, not neurosurgery, and short case length. In panel* ***C****, variables which significantly influenced the model toward a positive delirium prediction include not oriented to place, older age, ASA class 4, unable to rate pain using numeric assessment scale, high pressure ulcer risk, unable to spell ‘WORLD’ backwards, and high fall risk. Abbreviations: ASA, American Society of Anesthesiologists; ICD-10, International Classification of Diseases, 10th revision; ICD-10 F00-F99, mental and behavioral disorders; kg, kilograms; ICD-10 Z00-Z99, factors influencing health status and contact with health services; ICD-10 G00-G99, diseases of the nervous system; ERAS, enhanced recovery after surgery; ICD-10 C00-D48, neoplasms; ICD-10 I00-I99, diseases of the circulatory system; ICD-10 N00-N99, disease of the genitourinary system*

In the test dataset, the AUC-ROC was 0.840 (95% CI 0.825–0.855 by CV) and 0.841 (95% CI 0.816–0.863) by DL for Neural Network (Table 2, Fig. 2A.). AUC-ROC was 0.852 (95% CI 0.839–0.865) by CV and 0.851 (95% CI 0.827–0.874) by DL for XGBoost (Table 2, Fig. 2A.). Under the optimal threshold, Neural Network achieved a mean sensitivity of 72.9% (95% CI 69.1–76.7%), mean specificity of 77.5% (95% CI 76.2–78.7%), and mean positive likelihood ratio of 3.25 (95% CI 3.03–3.47). XGBoost achieved a mean sensitivity of 80.6% (95% CI 77.1–84.1%), mean specificity of 73.7% (95% CI 72.4–74.9%), and mean positive likelihood ratio of 3.08 (95% CI 2.87–3.29). Additional model characteristics and thresholds used for calculation of reported performance metrics are shown in Table 2. The 20 variables (out of the total pool of 115 variables) with the highest impact on XGBoost outcome prediction are pictured in Fig. 3A. Figure 3B and C demonstrate the XGBoost algorithm’s decision path for two individual patients. A calibration plot for each of the models is pictured in Fig. 2B. Neural Network, XGBoost, and ML hybrid models demonstrated excellent calibration, while calibration of the clinician-guided and AWOL-S models was moderate; they tended to overestimate delirium risk in those already at highest risk. We performed two sensitivity analyses: one including patients age 65 and older, and a second to further examine the impact of including neurosurgery patients in the broad population model on delirium prediction. In both patient subgroups, AUC-ROC of the XGBoost model did not change significantly (65+ AUC-ROC 0.821 [95% CI 0.763–0.879] by CV and 0.820 [95% CI 0.788–0.851] by DL; neurosurgery excluded AUC-ROC 0.840 [95% CI 0.808–0.871) by CV and 0.837 [95% CI 0.807–0.867] by DL) (Additional file 2, Figs. S1.2 and S2.2, Tables S2.2 and S2.2).

**Table 2:** Model Characteristics

***Model***	***Cutoff Value***	***AUC-ROC*** ***[95% CI]***	***Sensitivity*** ***[95% CI]***	***Specificity*** ***[95% CI]***	***PLR*** ***[95% CI]***	***PPV*** ***[95% CI]***	***NPV*** ***[95% CI]***
**Neural Network**	0.05	CV: 0.840 [0.825–0.855] DL: 0.841 [0.816–0.863]	72.9% [69.1–76.7%]	77.5% [76.2–78.7%]	3.25 [3.03–3.47]	15.1% [14.2–16.0%]	98.1% [97.9–98.4%]
**XGBoost**	0.25	CV: 0.852 [0.839–0.865] DL: 0.851 [0.827–0.874]	80.6% [77.1–84.1%]	73.7% [72.4–74.9%]	3.08 [2.87–3.29]	14.4% [13.5–15.3%]	98.6% [98.3–98.8%]
**Clinician-Guided Regression**	0.05	CV: 0.746 [0.718–0.775] DL: 0.763 [0.734–0.793]	69.1% [62.9–75.4%]	65.5% [64.3–66.7%]	2.01 [1.79–2.23]	9.0% [7.2–10.9%]	97.4% [96.9–98.0%
**ML Hybrid Regression**	0.32	CV: 0.810 [0.787–0.832] DL: 0.824 [0.800–0.849]	74.7% [69.8–79.6%]	73.5% [72.1–74.9%]	2.84 [2.46–3.09]	13.9% [12.7–15.1%]	98.1% [97.7–98.4%]
**AWOL-S**^**a**^	0.05	DL: 0.762 [0.713–0.812]	78.2% [66.0–89.3%]	60.0% [57.0–63.0%]	1.95 [1.62–2.28]	9.4% [6.8–12.3%]	98.1% [96.8–99.1%]

*Abbreviations: CI* confidence interval, *CV* cross validation, *DL* DeLong’s method

^***a***^AWOL-S is pre-validated, therefore cross validation was not performed to derive confidence intervals

The clinician-guided logistic regression model had an AUC-ROC of 0.746 (95% CI 0.718–0.775) by CV and 0.763 (95% CI 0.734–0.793) by DL, mean sensitivity 69.1% (95% CI 62.9–75.4%), mean specificity 65.5% (95% CI 64.3–66.7%), and mean positive likelihood ratio 2.01 (95% CI 1.79–2.23) (Table 2, Fig. 2A.). The ML hybrid model had an AUC-ROC of 0.810 (95% CI 0.787–0.832) by CV and 0.824 (95% CI 0.800–0.849) by DL, mean sensitivity of 74.7% (95% CI 69.8–79.6%), mean specificity of 73.5% (95% CI 72.1–74.9%), and mean positive likelihood ratio of 2.84 (95% CI 2.46–3.09) (Table 2, Fig. 2A.). Coefficients and odds ratios from the two regressions are available in Additional file 1, Tables S3 and S4. AWOL-S had an AUC-ROC of 0.762 (95% CI 0.713–0.812 by DL), mean sensitivity of 78.2% (95% CI 66.0–89.3%), mean specificity of 60.0% (95% CI 57.0–63.0%), and mean positive likelihood ratio of 1.95 (95% CI 1.62–2.28 (Table 2, Fig. 2A.).

## Discussion

We developed and internally validated two ML-derived risk prediction models which used preoperative data available in the EHR prior to the start of surgery to predict incident postoperative delirium in a broad surgical patient population. ML models offer better performance than traditional clinician-based regression models, both in this population and by comparison to published literature [40–42], implying that they could be used to more efficiently direct resources to patients at high risk compared with existing models [15] and with a clinician-guided logistic regression model derived on the same data. Further, a hybrid approach, using ML to select variables which were then input into a multivariable logistic regression, performed better than the purely clinician-guided approach.

Both ML-derived models achieved high AUC-ROCs (0.841 for Neural Net and 0.851 for XGBoost), similar to other published ML-derived risk prediction models [17, 43], and better than many risk prediction models specific for postoperative delirium [23, 24]. As opposed to most previously reported POD risk prediction models that focus on one particular surgical population [44], this model attempts to predict delirium in a broad surgical population over a wide age range. The inclusion of such a broad patient population was intentional, to make this a pragmatic tool for implementation in the perioperative setting. When compared to a simplified regression approach in an overlapping nonspecific perioperative population from our institution, ML models offer substantial improvement in discrimination [15]. Performance did not change significantly when models were rerun in patients over the age of 65 only, or when neurosurgical patients were excluded, suggesting the models are robust to different specifications of the underlying derivation population.

**Fig. 4:**
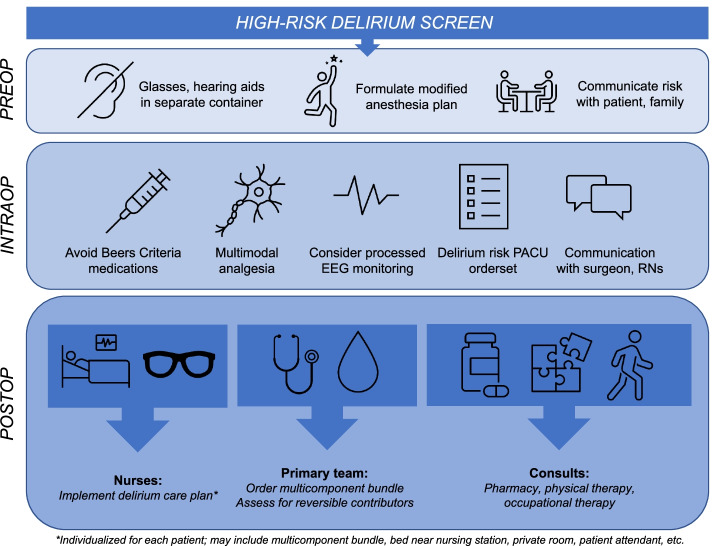
Role of automated delirium screen in our institution’s postoperative delirium prevention care pathway. *Figure legend*: A high-risk delirium screen triggers a set of care modifications in the preoperative, intraoperative, and postoperative phases of care. Particularly in the postoperative phase, these modifications require time and input from busy clinicians

Providing optimal care to patients at risk for delirium is a resource-intensive process which includes implementing measures such as multicomponent non-pharmacologic nursing care bundles and consultations from busy clinicians including pharmacists and rehabilitation professionals, whose time is a limited resource. A schematic depicting how this screening tool would be used in our healthcare system is pictured in Fig. 4, which also highlights the extensive resources automatically triggered by a high-risk delirium screen in our institution’s existing delirium prevention pathway [14, 45]. The care interventions were modeled after available guidelines for the prevention of postoperative delirium [1, 16, 46–48]. Recommendations common to nearly all these guidelines include performing preoperative cognitive screens and avoidance of high-risk medications. Postoperative components of our pathway, including use of multicomponent bundles, treatment of underlying causes, early mobility, and medication review, are recommended by those guidelines addressing care in the postoperative period [1, 16, 46, 47]. Identification of the patients at highest risk of developing delirium is the critical step to patient entry into the pathway. Thus, by improving the performance of delirium screening models, we could not only better target these resources to benefit the highest risk patients, but also potentially improve healthcare value. Next steps needed to operationalize the model would include prospective and external validation of the model followed by integration of the model into the EHR for real-time use; such real-time applications of ML models have been previously described [19], demonstrating potential feasibility of this approach.

Reporting model discrimination as the sole evaluation metric of a model’s performance is a commonly cited weakness of ML studies [49], since other measures of performance which take prevalence into account may be more indicative of clinical applicability and importance. Both of our ML models provide similar discrimination, but we also report other model characteristics (i.e., sensitivity, positive likelihood ratio, and positive predictive value) to address the potential for clinical applicability of our model. XGBoost and Neural Network both have high positive likelihood ratios, which would allow resource-intensive interventions to be directed to patients at high risk for developing delirium. We selected thresholds to optimize sensitivity and positive likelihood ratio, rather than positive predictive value, since positive predictive value is more subject to disease prevalence, and the overall prevalence of delirium is small in our population. The low prevalence of delirium compromised the positive predictive value somewhat. Further, we took steps to maximize interpretability of the XGBoost model by using the open-source SHAP package to visualize the global predictors that were deemed important by the algorithm as well as its individual, patient-centered decision approach to delirium prediction. These types of visualizations are helpful to increase clinician confidence in ML-derived predictions if they are to be used to augment clinical decision making [50]. The 20 most influential features identified by the XGBoost model generally align well with well-known risk factors for delirium, as discussed further below, lending credibility to the model’s prediction.

Not surprisingly, both ML models perform better than a traditional logistic regression analysis using delirium risk predictors selected from the 20-predictor model derived from multivariable analysis of the multi-institutional ACS-NSQIP dataset [37]. The ML hybrid model using the predictors derived from the XGBoost feature importance summary also outperforms the expert clinician regression model. This result suggests that ML-derived models are capable of uncovering higher-order interactions between variables that are difficult to identify using traditional regression approaches. Most of the predictors recovered by XGBoost (e.g., age, surgery type, cognitive impairment, comorbidities) are consistent with known predictors of POD [37, 51, 52]. Protective factors against delirium not frequently described but important to the model include having private insurance and a self-reported history of alcohol use, and a self-reported history of recent falls. It is likely that such features reflect the existence of associations which are not otherwise accounted for by the model. For example, those individuals with private insurance may have a higher socioeconomic status and/or education level. Patients capable of self-reporting falls may have better cognitive function when compared with those who are unable to do so. While alcohol consumption is a well-known risk factor for delirium [53], self-reported alcohol consumption has been associated with better functional outcomes including lower frailty in females [54] and lower likelihood of mobility limitation or arm function limitation independent of muscle strength in older men [55]. Other authors have suggested that the protective effect of moderate alcohol use is explained by social or lifestyle factors [55]; these may not be captured by other terms in the ML model. However, the exact mechanisms underlying these associations are unknown, and will need to be confirmed by further studies.

There are important limitations to consider when interpreting our findings. First, NuDESC ≥2 was used as the definition of delirium for patients on acute care wards. NuDESC is a delirium screening (not diagnostic) tool that was originally developed in a medical patient population [28]. It has been reported to have a wide sensitivity range to detect delirium in older surgical patients on the wards and in the post-anesthesia care unit, but specificity in surgical patients is reported to be 80% or greater [56, 57]. The high specificity of NuDESC in surgical patients suggests that the more severe, most clinically relevant cases, are detected. Despite reasonable concern for both false negative (i.e., undercounting the delirium outcome due to low sensitivity) and false positive (i.e., patients screening positive with NuDESC would not have delirium when formally assessed) results, it was necessary to take a pragmatic approach using the delirium screening procedures in place in our institution, due to the large training dataset needed to conduct this machine learning analysis. Comparison of our study to a recently published study by Racine, et al. [58] illustrates this point. This study evaluated the performance of five ML algorithms to predict POD in a much smaller group of surgical patients (560 older adults from an existing dataset). ML algorithms had a reported AUC-ROC 0.53–0.71, which was not superior to the logistic regression model reported in the study, despite using an in-person examination by experienced interviewers with the Confusion Assessment Method [59] and medical chart review to detect delirium. This comparison highlights the importance of an adequate training dataset size [60], among other things, to conduct high-quality machine learning analyses. Even with the large sample size in our study, discrimination and other aspects of model performance such as positive predictive value and calibration would likely be further improved with even more data and a larger number of events per predictor because our outcomes occur rarely [61].

Additional limitations include the lower POD rate (5.3%) in our population, which likely reflects inclusion of a younger population and all types of surgeries including those known to be associated with lower risk of delirium (i.e., gynecologic, urologic, plastics), in addition to robust delirium prevention procedures in our hospital system. When the analysis is excluded to patient age 65 and over the POD rate increases to 7.7%, which remains lower than commonly reported POD rates, likely still reflecting inclusion of patients having lower-risk surgery and staying in the hospital only overnight in our study population. Patients with no POD assessments were excluded from the analysis; it is possible that missingness is non-random for these patients. The fact that this was a single center study may introduce bias and limit generalizability. We conducted a sensitivity analysis to determine whether the high prevalence of neurosurgical patients in our population may have influenced the model, as this population tends to be at high risk of delirium despite younger age and fewer medical comorbidities. We found that the discrimination of the machine learning models was not significantly affected by exclusion of the neurosurgical population, suggesting that the model is able to conclude on delirium risk based on other risk features. In addition, there were limitations to gathering certain types of data from the EHR at our institution. Laboratory data is a good example; many of our patients either have no preoperative laboratory data available, or we are unable to easily extract this data from the EHR because results are housed in a scanned text report from an outside facility. There may also be potentially important delirium risk predictors (e.g., frailty indices) that are not included in our model because they are not routinely part of the preoperative examination at our institution. Expansion of the terms included in the ML model and external validation on a multicenter dataset would help to address these shortcomings.

## Conclusion

We developed and internally validated two ML-derived models that predict POD in a broad perioperative population using pragmatically collected EHR data. The XGBoost model offers the ability to understand the most impactful predictors and the process by which the algorithm arrives at a prediction for an individual patient. A ML-hybrid approach outperformed a clinician-guided logistic regression, suggesting that ML has the potential to uncover POD predictors that were previously overlooked by clinicians. As real-time clinical implementation of ML models becomes increasingly feasible, POD prediction using ML -- allowing targeted resource direction toward patients at the highest risk -- may be an important focus for improving perioperative care.

## Supplementary Information

**Additional file 1 : Supplementary Table S1**: List of variables included in the machine learning models. **Supplementary Table S2**: Comparison of the most important variables chosen by XGBoost and Neural Network. **Supplementary Table S3**: Multivariable logistic regression using variables selected by expert clinicians. **Supplementary Table S4**: Multivariable logistic regression using variables chosen by the XGBoost algorithm.

**Additional file 2 **Sensitivity Analyses. 1. Sensitivity Analysis in Patients Age 65 and Over. **Supplementary Figure S1.1.**: Inclusion Flow Diagram. **Supplementary Table S1.1**: Baseline Demographics. **Supplementary Figure S1.2:** Receiver Operating Characteristic (ROC) Curve For 5 Models. **Supplementary Table S1.2:** Comparison of Model Characteristics. **Supplementary Figure S1.3:** Feature Importance Summary of XGBoost Model. **Supplementary Table S1.3:** Comparison of Most Important Variables Chosen by XGBoost And Neural Network. **Supplementary Table S1.4:** Multivariable Logistic Regression Using Variables Selected by Expert Clinicians. **Supplementary Table S1.5**: Multivariable Logistic Regression Using Variables Chosen by The XGBoost Algorithm. 2. Sensitivity Analysis with Neurosurgery Patients Excluded. **Supplementary Figure S2.1.**: Inclusion Flow Diagram. **Supplementary Table S2.1**: Baseline Demographics. **Supplementary Figure S2.2:** Receiver Operating Characteristic (ROC) Curve For 5 Models. **Supplementary Table S2.2:** Confidence Intervals Of AUC-ROC. **Supplementary Figure S2.3:** Feature Importance Summary of XGBoost Model. **Supplementary Table S2.3:** Comparison of Most Important Variables Chosen by XGBoost And Neural Network. **Supplementary Table S2.4:** Multivariable Logistic Regression Using Variables Selected by Expert Clinicians. **Supplementary Table S2.5**: Multivariable Logistic Regression Using Variables Chosen by The XGBoost Algorithm.

## Data Availability

Data cannot be shared publicly because it contains potentially identifiable protected health information. For researchers who meet criteria to access confidential data, the process for obtaining access to such data is regulated by the University of California, as detailed at the following URL: https://data.ucsf.edu/cdrp/research.

## Abbreviations

POD: Postoperative delirium
ML: Machine learning
EHR: Electronic health record
XGBoost: EXtreme Gradient Boosting
AWOL-S: Delirium risk stratification tool using Age, ability to spell ‘World’ backward, Orientation to place, iLlness severity score, and Surgical risk
AUC-ROC: Area under the Receiver Operating Characteristic Curve
NuDESC: Nursing Delirium Screening Scale
CAM-ICU: Confusion Assessment Method for the Intensive Care Unit
ACS-NSQIP: American College of Surgeons National Surgical Quality Improvement Program
CI: Confidence interval
CV: 10-fold cross validation
DL: DeLong’s method
