# Supplementary material for: Postoperative delirium prediction using machine learning models and preoperative electronic health record data
Source: BMC Anesthesiol. 2022 Jan 3;22:8. doi: 10.1186/s12871-021-01543-y (PMC8722098; doi:10.1186/s12871-021-01543-y)
Supplement: Supplementary file 1 — Additional file 1 : Supplementary Table S1: List of variables included in the machine learning models. Supplementary Table S2: Comparison of the most important variables chosen by XGBoost and Neural Network. Supplementary Table S3: Multivariable logistic regression using variables selected by expert clinicians. Supplementary Table S4: Multivariable logistic regression using variables chosen by the XGBoost algorithm. [file 12871_2021_1543_MOESM1_ESM.docx]

**Supplementary Content – Additional File 1**

**Supplementary Table S1**: List of variables included in the machine learning models

**Supplementary Table S2**: Comparison of the most important variables chosen by XGBoost and Neural Network

**Supplementary Table S3**: Multivariable logistic regression using variables selected by expert clinicians

**Supplementary Table** **S4**: Multivariable logistic regression using variables chosen by the XGBoost algorithm

**Supplementary Table S1**: List of variables included in the machine learning models

| ***Category*** | ***Variable*** |
| --- | --- |
| Patient characteristics and demographics | Age  Gender  Race  Ethnicity  Primary language  Interpreter needed  Marital status  Insurance type  Need for transportation  Food insecurity  Financial resource strain  Emergency contact available  Admission status  Admission source  Billing source  Preoperative location |
| Medical history | Allergies  Number of allergies  Number of severe allergies  Past medical history (ICD 10 codes)  Number of prior anesthetic encounters in the past 3 years  Smoking status  Alcohol use  Substance use  Prior admission 30 days  Prior admission 90 days  ASA Class |
| Surgical characteristics | Day of surgery  Month of year  Procedure name  ERAS patient (yes/no)  Predicted case length  Case location  Time of induction  Delayed incision (yes/no)  Emergent case  Case classification  Admitting service  Surgical service  Planned anesthetic type |
| Preoperative nursing assessments | Learning preference  Barriers to learning (visual, hearing, cognitive, etc)  Type of pain assessment (ex: numeric, faces, CPOT)  Pain or discomfort  Pain level (0-10)  Acceptable level of pain  Type of pain  Location of pain  Area of pain  Effect of pain on daily activities  Multiple areas of pain (yes/no)  Pain relieving factors  Pain treatment strategies  Thermoregulation interventions  Respiratory interventions  Ability to spell ‘WORLD’ backwards  Illness severity score  Orientation to place  Access to transportation  ADL: feeding  ADL: grooming  ADL: bathing  ADL: dressing  ADL: bed transferring  Weakness in arms  Weakness in legs  Bowel and bladder habits  Vision issues  Hearing issues (R ear)  Hearing issues (L ear)  Mobility issues  Elimination issues  History of falls  Schmid Fall score  Braden scale  Fall risk stratification (0-5)  Problem documented: Neuro  Problem documented: Cardiac  Problem documented: Skin  Problem documented: Musculoskeletal  Problem documented: Genitourinary  Problem documented: Anus and rectum  Problem documented: HEENT  Problem documented: Psychosocial  Violence abuse assessment  Social worker consultation needed  Suicide risk  Unplanned weight loss  Difficulty chewing  Difficulty swallowing  Tube feeds  Total parenteral nutrition  Presence of pressure ulcer  Non healing wound  Dietician consultation needed  Oxygen device  Braces / devices / sensory aids  Sleep habit details  Glasgow Coma Score |
| Vital signs | Temperature  Pulse rate  Source of pulse  Respirations  Oxygen saturation  Blood pressure  Source of blood pressure  Weight (kg)  BMI  Height (cm)  Body surface area |
| Labs | Glucose |
| ***Abbreviations****: ICD 10, International Classification of Diseases, 10^th^ revision; ASA, American Society of Anesthesiologists; ERAS, enhanced recovery after surgery; CPOT, critical care pain observation tool; ADL, activities of daily living; HEENT, head eyes ears nose throat; kg, kilogram; BMI, body mass index; cm, centimeter* | |

**Supplementary Table S2**: Comparison of most important variables chosen by XGBoost (left) and Neural Network (right). Variables are listed alphabetically. For XGBoost, direction of effect is noted in a separate column. Variables denoted by * are unique to the respective model.

| ***XGBoost*** | | ***Neural Network*** |
| --- | --- | --- |
| *Variable* | *Direction of effect* | *Variable* |
| Ability to answer violence/abuse assessment | Yes = Protective factor | Ability to answer violence/abuse assessment |
| Ability to spell ‘WORLD’ backwards | Yes = Protective | Ability to spell ‘WORLD’ backwards |
| Age | Higher = Risk factor | Higher age |
| ASA Class | Higher = Risk factor | ASA Class |
| Braden Scale | Lower score (Higher pressure ulcer risk) = Risk factor | Braden Scale |
| Fall risk | Low fall risk = Protective factor | ERAS (enhanced recovery after surgery) case* |
| History of alcohol use | Does not self-report alcohol use = Risk factor | Fall risk |
| History of mental and behavioral disorders (ICD10 group F00-F99) | Yes = Risk factor | History of alcohol use |
| Inpatient status | Yes = Risk factor  No = Protective factor | History of diseases in the cardiovascular system (ICD10 group I00-I99)* |
| Medicare/Medical payor* | Yes = Risk factor | History of diseases in the nervous system (ICD10 group G00-G99) |
| Name of surgical procedure ending in the phrase ‘-plasty’ | Yes = Protective factor | History of mental and behavioral disorders (ICD10 group F00-F99) |
| Neurological Surgery Service | Yes = Risk factor | Inpatient status |
| Number of prior anesthesia encounters in past 3 years* | Higher = Risk factor | Marital status* |
| Other factors influencing health status and contact with health services (ICD10 group Z00-Z99) | Yes = Protective factor | Name of surgical procedure ending in the phrase ‘-plasty’ |
| Private Health Insurance | Yes = Protective factor | Neurological Surgery Service |
| Predicted Case Length | Longer case = Risk factor | Private Health Insurance |
| Use of numeric scale for preoperative pain assessment | Yes = Protective factor | Prior hospitalization within the past 90 days* |
| Weight | Lower = Risk factor | Weight |

**Supplementary Table S3**: Multivariable logistic regression using variables selected by expert clinicians^1^

| ***Multivariable Logistic Regression using Expert-Clinician Variables*** | ***Beta*** | ***Odds Ratio***  ***[95% CI]*** | ***p-value*** |
| --- | --- | --- | --- |
| Age | 0.030 | 1.03  [1.026-1.035] | <0.001 |
| Male | -0.180 | 0.835  [0.743-0.938] | 0.002 |
| ASA Class ≥ 3 | 0.751 | 2.12 [1.85-2.44] | <0.001 |
| Emergency case | 0.757 | 2.13 [1.88-2.41] | <0.001 |
| High risk surgical service  (Cardiac, Thoracic, Vascular, Neurological) | 0.850 | 2.34 [2.08-2.64] | <0.001 |
| Primary language not English | -0.230 | 0.795  [0.648-0.968] | 0.025 |
| Pre-existing cognitive impairment  (unable to spell ‘WORLD’ backwards) | 1.001 | 2.72  [2.31-3.19] | <0.001 |
| Dependency on ADLs | 0.370 | 1.45 [1.07-1.92] | 0.012 |
| Visual or hearing impairment | 0.273 | 1.31 [0.913-1.86] | 0.132 |
| Patient-reported history of falls | 0.149 | 1.16 [0.854-1.33] | 0.329 |
| Evidence of malnutrition (tube feeds, TPN, pressure ulcers) | 0.177 | 1.19 [0.766-1.82] | 0.422 |
| History of diabetes (T1DM and T2DM) | 0.062 | 1.06 [0.929-1.22] | 0.368 |
| History of heart failure (ICD-10 I50) | 0.069 | 1.07 [0.854-1.33] | 0.543 |
| History of CKD in all patients (ICD-10 N18) | -0.277 | 0.758  [0.626-0.913] | 0.004 |
| *History of CKD in non-transplant patients (ICD-10 N18)^a^* | *0.550* | *1.73 [0.710-3.97]* | *0.205* |
| History of dyspnea (ICD-10 R06) | -0.408 | 0.665  [0.392-1.06] | 0.104 |
| History of sepsis (ICD-10 A41 or R78) | 0.522 | 1.68 [1.08-2.53] | 0.016 |
| Patient reported history of smoking (Yes, Prior, Quit) | 0.256 | 1.29 [1.14-1.46] | <0.001 |
| ***Abbreviations:*** *CI, confidence interval; ASA, American Society of Anesthesiologists; ADLs, activities of daily living; TPN, total parenteral nutrition; T1DM, type 1 diabetes mellitus; T2DM, type 2 diabetes mellitus; ICD-10, International Classification of Diseases version 10; CKD, chronic kidney disease* | | | |

^1^Berian JR, Zhou L, Russell MM, et al. Postoperative Delirium as a Target for Surgical Quality Improvement. *Ann Surg*. 2018;268(1):93-99. doi:10.1097/SLA.0000000000002436.

^a^Performed only during post-hoc sensitivity analysis, not used in model evaluation

**Supplementary Table** **S4**: Multivariable logistic regression using variables chosen by the XGBoost algorithm*

| ***Multivariable Logistic Regression using Machine-Learning Derived Variables*** | ***Beta*** | ***Odds Ratio [95% CI]*** | ***p-value*** |
| --- | --- | --- | --- |
| Age | 0.025 | 1.02 [1.02-1.03] | <0.001 |
| BMI | -0.022 | 0.979  [0.970-0.987] | <0.001 |
| ASA Class ≥ 3 | 0.636 | 1.88 [1.63-2.18] | <0.001 |
| Neurological surgery | 1.01 | 2.75 [2.39-3.15] | <0.001 |
| Inpatient status | 1.69 | 5.42 [3.44-9.20] | <0.001 |
| Private insurance | -0.335 | 0.715  [0.613-0.831] | <0.001 |
| History of neurologic disease (ICD10 category G00-G99) | 0.049 | 1.05 [0.976-1.13] | 0.187 |
| Factors influencing health status (ICD10 category Z00-Z99) | -0.123 | 0.884  [0.831-0.938] | <0.001 |
| History of mental and behavioral disorders (ICD10 category F00-F99) | 0.215 | 1.24 [1.16-1.32] | <0.001 |
| Patient reported history of falls | -0.223 | 0.800  [0.579-1.08] | 0.164 |
| Completed violence and abuse assessment | -0.421 | 0.657  [0.555-0.780] | <0.001 |
| Physician referral for procedure | -0.562 | 0.570  [0501-0.649] | <0.001 |
| Patient reported history of alcohol use  (Yes, Not Currently) | -0.163 | 0.850  [0.739-0.974] | 0.020 |
| Braden Scale (pressure ulcer risk; higher number = lower risk) | -0.204 | 0.815 [0.798-0.832] | <0.001 |
| Higher preoperative numerical pain rating | -0.159 | 0.853  [0.731-0.992] | 0.041 |
| Pre-existing cognitive impairment  (unable to spell ‘WORLD’ backwards) | 0.665 | 1.95 [1.64-2.30] | <0.001 |
| Predicted surgical case length at time of booking (minutes) | 0.0004 | 1.00 [0.999-1.00] | 0.113 |
| Procedures ending in the phrase ‘-plasty’ | -0.702 | 0.496  [0.386-0.628] | <0.001 |
| ***Abbreviations****: CI, confidence interval; BMI, body mass index; ASA, American Society of Anesthesiologists; ICD-10, International Classification of Diseases, 10^th^ Revision* | | | |

** The model was developed from an iteration of the XGBoost algorithm using the following hyper-parameters in XGBoost model development: learning rate=0.05, maximum tree depth=7, minimum child weight=7, number of estimators=150, scaled positive weight=10.*
