# Supplementary material for: Postoperative delirium prediction using machine learning models and preoperative electronic health record data
Source: BMC Anesthesiol. 2022 Jan 3;22:8. doi: 10.1186/s12871-021-01543-y (PMC8722098; doi:10.1186/s12871-021-01543-y)
Supplement: Supplementary file 2 — Additional file 2 Sensitivity Analyses. 1. Sensitivity Analysis in Patients Age 65 and Over. Supplementary Figure S1.1.: Inclusion Flow Diagram. Supplementary Table S1.1: Baseline Demographics. Supplementary Figure S1.2: Receiver Operating Characteristic (ROC) Curve For 5 Models. Supplementary Table S1.2: Comparison of Model Characteristics. Supplementary Figure S1.3: Feature Importance Summary of XGBoost Model. Supplementary Table S1.3: Comparison of Most Important Variables Chosen by XGBoost And Neural Network. Supplementary Table S1.4: Multivariable Logistic Regression Using Variables Selected by Expert Clinicians. Supplementary Table S1.5: Multivariable Logistic Regression Using Variables Chosen by The XGBoost Algorithm. 2. Sensitivity Analysis with Neurosurgery Patients Excluded. Supplementary Figure S2.1.: Inclusion Flow Diagram. Supplementary Table S2.1: Baseline Demographics. Supplementary Figure S2.2: Receiver Operating Characteristic (ROC) Curve For 5 Models. Supplementary Table S2.2: Confidence Intervals Of AUC-ROC. Supplementary Figure S2.3: Feature Importance Summary of XGBoost Model. Supplementary Table S2.3: Comparison of Most Important Variables Chosen by XGBoost And Neural Network. Supplementary Table S2.4: Multivariable Logistic Regression Using Variables Selected by Expert Clinicians. Supplementary Table S2.5: Multivariable Logistic Regression Using Variables Chosen by The XGBoost Algorithm. [file 12871_2021_1543_MOESM2_ESM.docx]

**­Supplementary Content: Additional File 2, Sensitivity Analyses**

1. **Sensitivity Analysis in Patients Age 65 and Over**

**Supplementary Figure S1.1.**: Inclusion Flow Diagram

**Supplementary Table S1.1**: Baseline Demographics

**Supplementary Figure S1.2:** Receiver Operating Characteristic (ROC) Curve For 5 Models*

**Supplementary Table S1.2:** Comparison of Model Characteristics*

**Supplementary Figure S1.3:** Feature Importance Summary of XGBoost Model*

**Supplementary Table S1.3:** Comparison of Most Important Variables Chosen by XGBoost And Neural Network*

**Supplementary Table S1.4:** Multivariable Logistic Regression Using Variables Selected by Expert Clinicians

**Supplementary Table S1.5**: Multivariable Logistic Regression Using Variables Chosen by The XGBoost Algorithm*

1. **Sensitivity Analysis with Neurosurgery Patients Excluded**

**Supplementary Figure S2.1.**: Inclusion Flow Diagram

**Supplementary Table S2.1**: Baseline Demographics

**Supplementary Figure S2.2:** Receiver Operating Characteristic (ROC) Curve For 5 Models*

**Supplementary Table S2.2:** Confidence Intervals Of AUC-ROC*

**Supplementary Figure S2.3:** Feature Importance Summary of XGBoost Model*

**Supplementary Table S2.3:** Comparison of Most Important Variables Chosen by XGBoost And Neural Network*

**Supplementary Table S2.4:** Multivariable Logistic Regression Using Variables Selected by Expert Clinicians

**Supplementary Table S2.5**: Multivariable Logistic Regression Using Variables Chosen by The XGBoost Algorithm*

** The following hyper-parameters were used in XGBoost model development for subgroup analyses: learning rate=0.05, maximum tree depth=7, minimum child weight=7, number of estimators=150, scaled positive weight=10*

1. **Sensitivity Analysis: Patients Age 65 and Over**

**Supplementary Figure S1.1.**: Inclusion Flow Diagram

**
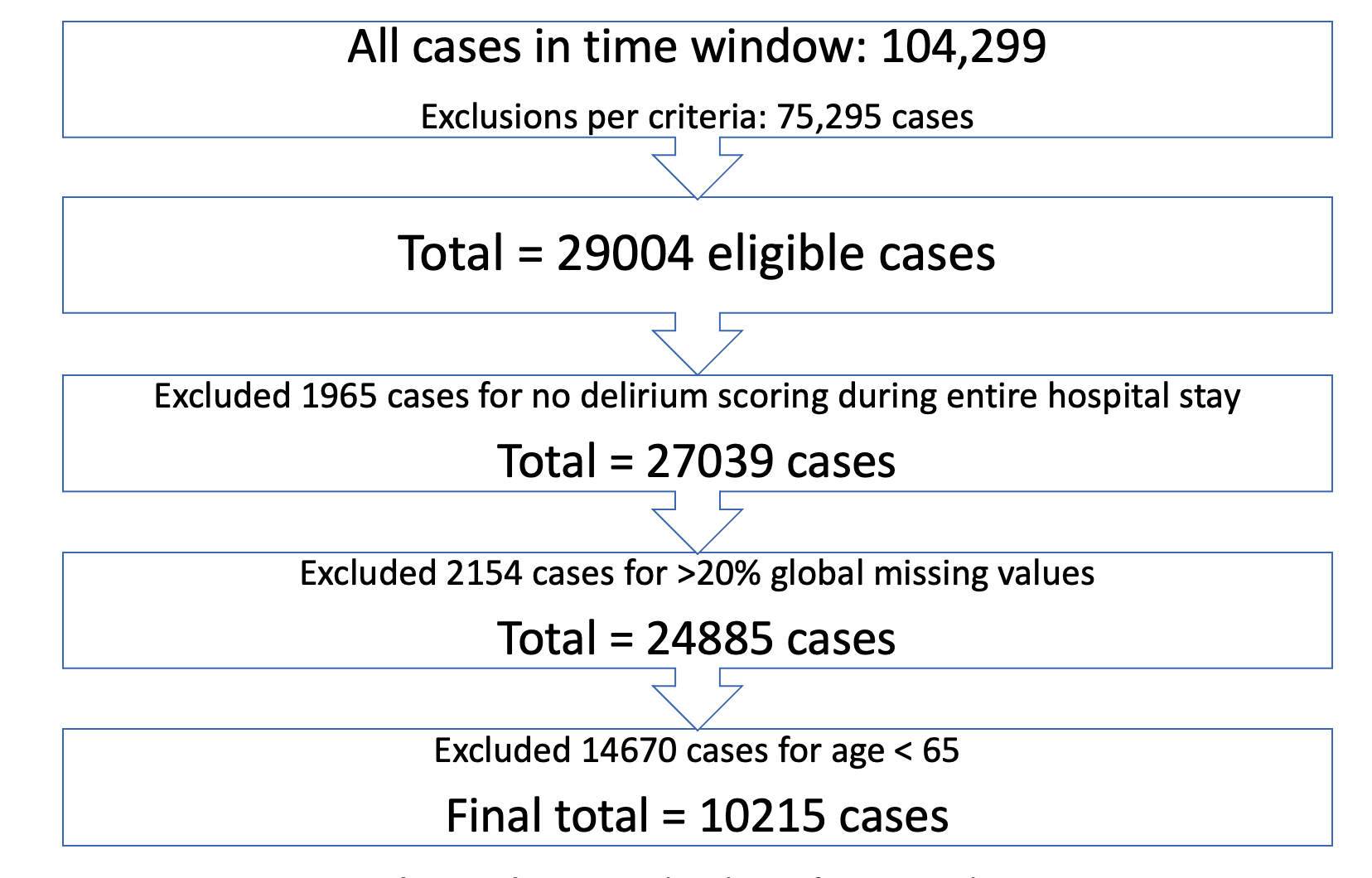
**

**Supplementary Table S1.1**: Baseline Demographics (Patients Age 65 and Over)

| ***Variable*** | ***All***  *(n=10,215)* | ***No Delirium***  *(n=9397)* | ***Delirium***  *(n=818)* | ***p-value*** | *% Missing* |  |
| --- | --- | --- | --- | --- | --- | --- |
| Age (mean (SD)) | 73.76 (6.53) | 73.51 (6.35) | 76.66 (7.74) | <0.001 | 0 |  |
| Male Gender (%) | 5361 (52.5) | 4969 (52.9) | 392 (47.9) | 0.022 | 0 |  |
| ASA Class (%) | | | | | 0.7 |  |
| 1 | 89 (0.9) | 87 (0.9) | 2 (0.2) | 0.071 |  |  |
| 2 | 4274 (42.2) | 4101 (44.0) | 173 (21.4) | <0.001 |  |  |
| 3 | 5311 (52.4) | 4745 (50.9) | 566 (70.0) | <0.001 |  |  |
| 4 | 462 (4.6) | 396 (4.2) | 66 (8.2) | <0.001 |  |  |
| Emergent Case (%) | 1133 (11.1) | 914 (9.7) | 219 (26.8) | <0.001 | 0 |  |
| Inpatient (%) | 9068 (88.8) | 8261 (87.9) | 807 (98.7) | <0.001 | 0 |  |
| Surgical Service (%) | | | | | 0 |  |
| General Surgery | 1764 (17.3) | 1630 (17.3) | 134 (16.4) | 0.515 |  |  |
| Neurological Surgery | 1486 (14.5) | 1259 (13.4) | 227 (27.8) | <0.001 |  |  |
| Thoracic Surgery | 248 (2.4) | 237 (2.5) | 11 (1.3) | 0.048 |  |  |
| Vascular Surgery | 723 (7.1) | 627 (6.7) | 96 (11.7) | <0.001 |  |  |
| Genito-Urologic Surgery | 1183 (11.6) | 1155 (12.3) | 28 (3.4) | <0.001 |  |  |
| Transplant Surgery | 239 (2.3) | 227 (2.4) | 12 (1.5) | 0.109 |  |  |
| Primary Language (%) | | | | | 0 |  |
| English | 239 (2.3) | 8420 (89.6) | 712 (87.0) | 0.026 |  |  |
| Spanish | 341 (3.3) | 318 (3.4) | 23 (2.8) | 0.440 |  |  |
| Chinese - Cantonese | 242 (2.4) | 206 (2.2) | 36 (4.4) | <0.001 |  |  |
| Unable to spell WORLD backwards (%) | 841 (20.5) | 669 (17.8) | 172 (50.7) | <0.001 | 0 |  |
| Not oriented to place (%) | 313 (7.6) | 221 (5.8) | 92 (27.2) | <0.001 | 0 |  |
| History of Diabetes (%) | 2407 (23.6) | 2137 (22.7) | 270 (33.0) | <0.001 | 0 |  |
| History of Chronic Kidney Disease (%) | 1041 (10.2) | 938 (10.0) | 103 (12.6) | 0.021 | 0 |  |
| History of Heart Failure (%) | 608 (6.0) | 529 (5.6) | 79 (9.7) | <0.001 | 0 |  |
| History of Smoking (%) | 245 (2.4) | 220 (2.3) | 25 (3.1) | 0.245 | 0 |  |
| *Abbreviations: SD, standard deviation; ASA, American Society of Anesthesiologists* | | | | | | |

**Supplementary Figure S1.2:** Receiver Operating Characteristic (ROC) Curve For 5 Models (Patients Age 65 and Over)

 
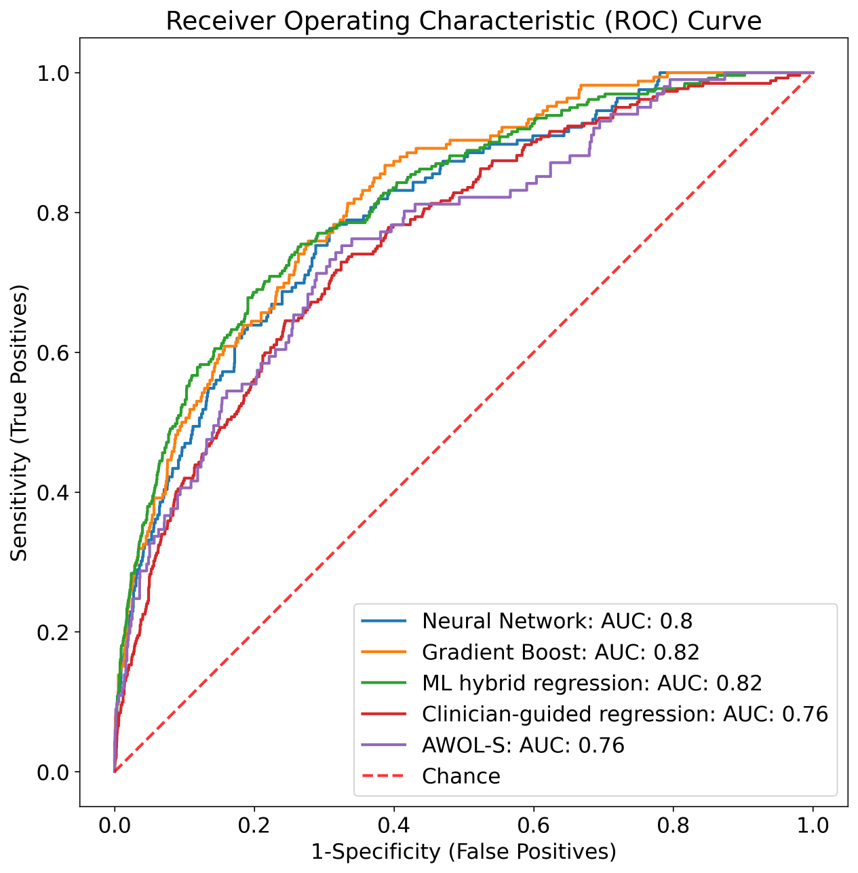


Neural Network: AUC: 0.80

XGBoost: AUC: 0.82

ML hybrid regression: AUC: 0.82

Clinician-guided regression: AUC: 0.76

AWOL-S: AUC: 0.76

Chance

**Supplementary Table S1.2: Comparison of Model Characteristics** (Patients Age 65 and Over)

| ***Model*** | ***AUC-ROC*** | ***At 90% Sensitivity Threshold:*** | ***At 90% Specificity Threshold:*** |
| --- | --- | --- | --- |
| Neural Network | CV 0.80 [0.75-0.86]  DL 0.80  [0.77-0.84] | Actual threshold: 4%  Specificity: 0.47 [0.44-0.5]  PPV: 0.13 [0.12-0.14]  NPV: 0.98 [0.97-0.99]  NNS: 1.2 | Actual threshold: 13%  Sensitivity: 0.48 [0.37-0.59]  PPV: 0.3 [0.23-0.36]  NPV: 0.95 [0.95-0.96]  NNS: 2.5 |
| XGBoost | CV 0.82 [0.76-0.88]  DL 0.82 [0.79-0.85] | Actual threshold: 17%  Specificity: 0.53 [0.51-0.56]  PPV: 0.15 [0.13-0.16]  NPV: 0.98 [0.97-1.0]  NNS: 1.2 | Actual threshold: 50%  Sensitivity: 0.46[0.41-0.52]  PPV: 0.31 [0.24-0.37]  NPV: 0.95 [0.95-0.95]  NNS: 2.6 |
| ML Hybrid Logistic Regression | CV 0.82 [0.78-0.86]  DL 0.82 [0.79-0.85] | Actual threshold: 4%  Specificity: 0.5[ 0.48-0.52]  PPV: 0.09 [0.07-0.11]  NPV: 0.93 [0.92-0.95]  NNS: 1.2 | Actual threshold: 18%  Sensitivity: 0.45[0.37-0.52]  PPV: 0.08 [0.04-0.13]  NPV: 0.92 [0.91-0.93]  NNS: 2.7 |
| Expert-Clinician Logistic Regression | CV 0.76 [0.71-0.81]  DL 0.76 [0.72-0.80] | Actual threshold: 4%  Specificity: 0.31 [0.28-0.34]  PPV: 0.11 [0.1-0.11]  NPV: 0.98 [0.96-0.99]  NNS: 1.2 | Actual threshold: 14%  Sensitivity: 0.37 [0.28-0.45]  PPV: 0.26 [0.19-0.32]  NPV: 0.94 [0.93-0.95]  NNS: 3.4 |
| AWOL-S | CV 0.76 [n/a]  DL 0.69 [0.63-0.76] | Actual threshold: 4%  Specificity: 0.22  PPV: 0.10  NPV: 0.96  NNS: 1.2 | Actual threshold: 14%  Sensitivity: 0.32  PPV: 0.32  NPV: 0.93  NNS: 4.2 |
| *Abbreviations: CV, cross validation; DL, DeLong’s Method; PPV, positive predictive value; NPV, negative predictive value; NNS, number needed to screen* | | | |

**Supplementary Figure S1.3:** Feature Importance Summary of XGBoost Model (Patients Age 65 and Over)

**
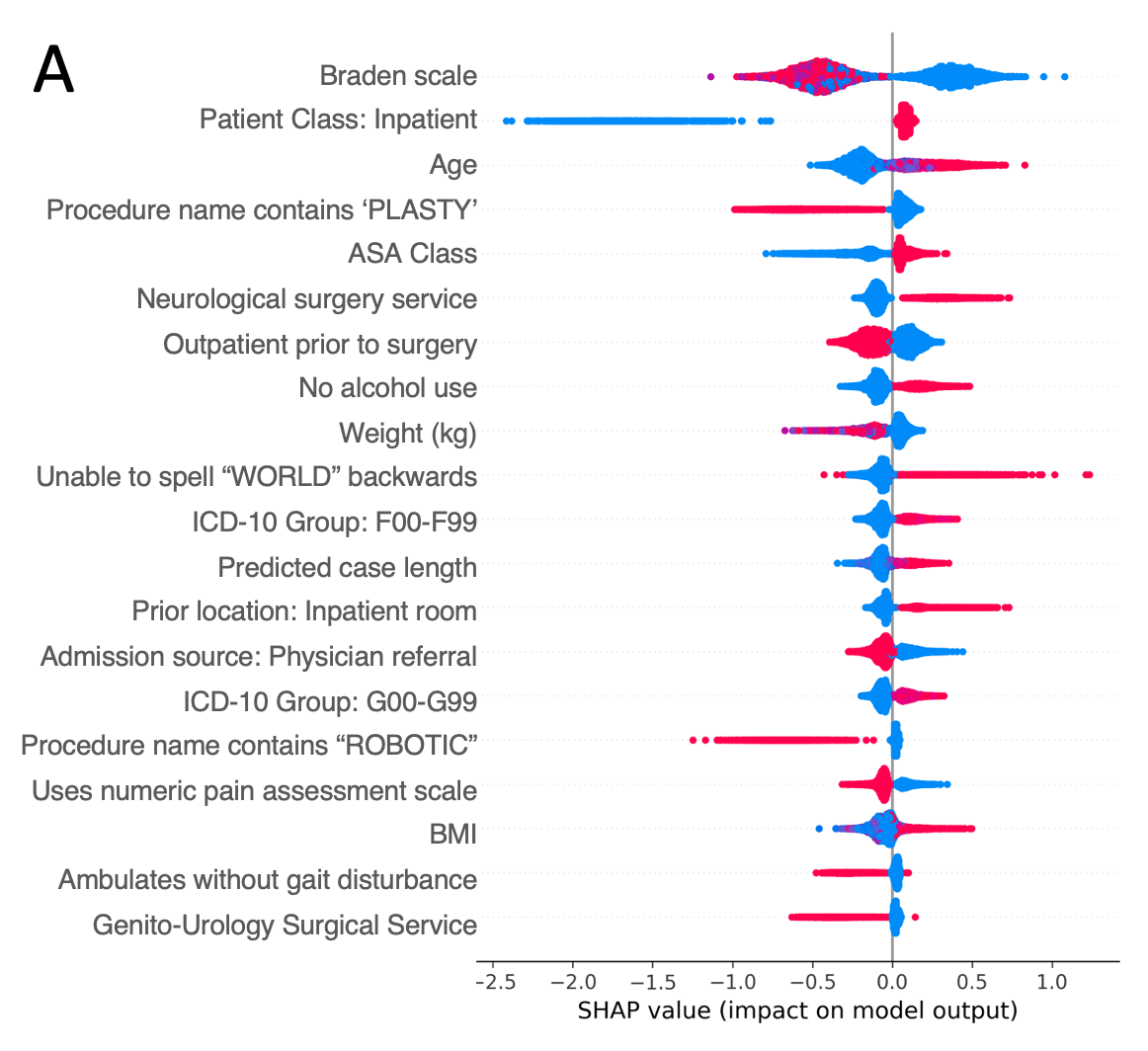
**

Top 20 most influential variables used by XGBoost (Patients Age 65 and Over). *Interpretation*: *Each dot represents an individual instance. Red dots indicate higher numeric value or yes/present for categorical variables, and blue dots indicate lower numeric value or no/absent. Dots on the right side of the y-axis indicate a higher impact on the model to predict delirium, and dots to the left of the y-axis indicate features that are protective against prediction of delirium. Dots with the same impact value are plotted on top of each other to create a vertical distribution.*

*Abbreviations: ASA, American Society of Anesthesiologists; kg, kilograms; ICD-10, International Classification of Diseases, 10th revision; ICD-10 F00-F99, mental and behavioral disorders; preop, preoperative; ICD-10 G00-G99, diseases of the nervous system; BMI, body mass index*

**Supplementary Table S1.3:** Comparison of Most Important Variables Chosen by XGBoost And Neural Network (Patients Age 65 and Over).

Comparison of most important variables chosen by XGBoost (left) and Neural Network (right).. Variables are listed alphabetically. For XGBoost, direction of effect is noted in a separate column. Variables denoted by * are unique to the respective model.

| **Important Variables by XGBoost** | | **Important Variables by Neural Net** |
| --- | --- | --- |
| *Variable* | *Direction of effect* | *Variable* |
| Ability to spell ‘WORLD’ backwards | No = Risk Factor | Ability to spell ‘WORLD’ backwards |
| Age* | Higher = Risk Factor | Acceptable Numerical Level of Pain* |
| Ambulates without gait disturbance* | Yes = Protective Factor | ASA Class |
| ASA Class | Higher = Risk Factor | Braden Scale |
| BMI* | Higher = Risk Factor | Emergent Case* |
| Braden Scale | High risk = Risk factor | Existing pain location* |
| Genito-Urology Surgical Service | Yes = Protective Factor | Existing preoperative musculoskeletal problem* |
| History of alcohol use * | No = Risk Factor | Fall Risk* |
| History of mental and behavioral disorders (ICD10 group F00-F99)* | Yes = Risk Factor | Genito-Urology Surgical Service |
| History of diseases in the nervous system (ICD10 group G00-G99)* | Yes = Risk Factor | Inpatient status (Patient class and Prior location) |
| Inpatient status (Patient class and Prior location) | Yes = Risk Factor | Name of surgical procedure containing the phrase ‘ABDOMINAL’ * |
| Name of surgical procedure ending in the phrase ‘-PLASTY’ | Yes = Protective Factor | Name of surgical procedure containing the phrase ‘AORTIC’ * |
| Name of surgical procedure containing the phrase ‘ROBOTIC’ * | Yes = Protective Factor | Name of surgical procedure ending in the phrase ‘-PLASTY’ |
| Neurological Surgery Service | Yes = Risk Factor | Neurological Surgery Service |
| Predicted Case Length* | Longer = Risk Factor | Referral Source for patient |
| Use of numeric scale for preoperative pain assessment* | Yes = Protective Factor |  |
| Referral source for patient (Physician Referral)* | Yes = Protective Factor |  |
| Weight (kg)* | Lower = Protective Factor |  |

**Supplementary Table S1.4:** Multivariable Logistic Regression Using Variables Selected by Expert Clinicians^1^ (Patients Age 65 and Over)

| ***^Multivariate Logistic Regression using Expert-Clinician Variables^*** | ***^Beta^*** | ***^Odds Ratio^***  ***^[95% CI]^*** | ***^p-value^*** |
| --- | --- | --- | --- |
| ^Age^ | ^0.0479^ | ^1.05 [1.038-1.060]^ | ^<0.001^ |
| ^Male^ | ^-0.334^ | ^0.72 [0.62-0.83]^ | ^<0.001^ |
| ^ASA Class > 3^ | ^0.655^ | ^1.93 [1.61-2.32]^ | ^<0.001^ |
| ^Emergency Case^ | ^0.637^ | ^1.89 [1.61-2.22]^ | ^<0.001^ |
| ^High risk surgical service^  ^(Cardiac, Thoracic, Vascular, Neurological)^ | ^0.794^ | ^2.21 [1.89-2.58]^ | ^<0.001^ |
| ^Primary Language not English^ | ^-0.190^ | ^0.83 [0.65-0.1.0]^ | ^0.11^ |
| ^Pre-existing cognitive impairment^  ^(unable to spell ‘WORLD’ backwards)^ | ^0.896^ | ^2.45 [1.99-3.00]^ | ^<0.001^ |
| ^Dependency on ADLs^ | ^0.311^ | ^1.37 [0.94-1.96]^ | ^0.098^ |
| ^Visual or hearing impairment^ | ^0.430^ | ^1.54 [1.00-2.32]^ | ^0.044^ |
| ^Patient-reported history of falls^ | ^0.172^ | ^1.19 [0.821-1.68]^ | ^0.35^ |
| ^Evidence of malnutrition (tube feeds, TPN, pressure ulcers)^ | ^0.023^ | ^1.02 [0.56-1.82]^ | ^0.94^ |
| ^History of diabetes (T1DM and T2DM)^ | ^0.230^ | ^1.26 [1.06-1.49]^ | ^<0.01^ |
| ^History of heart failure (ICD-10 I50)^ | ^0.086^ | ^1.09 [0.83-1.42]^ | ^0.53^ |
| ^History of CKD (ICD-10 N18)^ | ^-0.158^ | ^0.853 [0.7-1.08]^ | ^0.19^ |
| ^History of dyspnea (ICD-10 R06)^ | ^-0.367^ | ^0.69 [0.39-1.18]^ | ^0.21^ |
| ^History of sepsis (ICD-10 A41 or R78)^ | ^0.512^ | ^1.67 [0.85-3.03]^ | ^0.11^ |
| ^Patient reported history of smoking (Yes, Prior, Quit)^ | ^0.161^ | ^1.17 [1.00-1.38]^ | ^0.05^ |
| *^Abbreviations ASA, American Society of Anesthesiologists; ADL, activities of daily living; TPN, total parenteral nutrition; T1DM, type 1 diabetes mellitus; T2DM, type 2 diabetes mellitus; ICD-10, International Classification of Diseases, 10th ed^* | | | |

^1^Berian JR, Zhou L, Russell MM, et al. Postoperative Delirium as a Target for Surgical Quality Improvement. *Ann Surg*. 2018;268(1):93-99. doi:10.1097/SLA.0000000000002436.

**Supplementary Table S1.5**: Multivariable Logistic Regression Using Variables Chosen by The XGBoost Algorithm (Patients Age 65 and Over)

| ***Multivariate Logistic Regression using Machine-Learning Derived Variables*** | ***Beta*** | ***Odds Ratio [95% CI]*** | ***p-value*** |
| --- | --- | --- | --- |
| Age | 0.0368 | 1.04  [1.03-1.05] | <0.001 |
| BMI | 0.0064 | 1.00  [0.989-1.02] | 0.46 |
| Weight (kg) | -0.0095 | 0.99  [0.984-0.997] | 0.002 |
| ASA Class > 3 | 0.500 | 1.65  [1.37-2.00] | <0.001 |
| Inpatient Status | 1.87 | 6.47  [3.50-13.7] | <0.001 |
| Physician referral for procedure | -0.328 | 0.72  [0.61-0.86] | <0.001 |
| Braden scale  (Pressure ulcer risk, higher number = lower risk) | -0.194 | 0.82  [0.80-0.85] | <0.001 |
| Pre-existing cognitive impairment  (unable to spell ‘WORLD’ backwards) | 0.521 | 1.68  [1.35-2.09] | <0.001 |
| Patient reported history of alcohol use  (Yes, Not Currently) | -0.240 | 0.79  [0.65-0.94] | <0.01 |
| Ability to ambulate without gait issues | -0.233 | 0.79  [0.59-1.05] | 0.12 |
| Use of numerical pain scale to rate pain | -0.544 | 0.58  [0.49-0.68] | <0.001 |
| History of neurologic disease (ICD10 category G00-G99) | 0.072 | 1.07  [0.98-1.18] | 0.14 |
| History of mental and behavioral disorders (ICD10 category F00-F99) | 0.217 | 1.24  [1.13-1.36] | <0.001 |
| Predicted surgical case length at time of booking (minutes) | 0.00089 | 1.00  [1.00-1.00] | 0.011 |
| Procedures ending in the phrase ‘-plasty’ | 0.384 | 0.38  [0.28-0.52] | <0.001 |
| Procedures containing the phrase ‘ROBOTIC’ | 0.308 | 0.31  [0.14-0.59] | 0.10 |
| Neurological Surgery Service | 2.28 | 2.28  [1.88-2.76] | <0.001 |
| Genito-Urologic Surgery Service | 0.704 | 0.70  [0.45-1.05] | 0.0013 |
| *Abbreviations: BMI, body mass index; kg, kilograms; ASA, American Society of Anesthesiologists; ICD-10, International Classification of Diseases, 10^th^ ed.* | | | |

1. **Sensitivity Analysis: Neurosurgery Patients Excluded**

**Supplementary Figure S2.1**: Inclusion Flow Diagram (Neurosurgery Patients Excluded)

**Supplementary Table S2.1**: Baseline demographics (Neurosurgery Patients Excluded)

Delirium incidence = 4.3%

| ***Variable*** | ***No Delirium***  *(n=20220)* | ***Delirium***  *(n=926)* | ***p-value*** | *% Missing* |
| --- | --- | --- | --- | --- |
| Age (mean (SD)) | 73.76 (6.53) | 59.47 (15.31) | 69.23 (14.89) | <0.001 |
| Gender (%)­­ |  |  |  | 0 |
| Male | 5361 (52.5) | 10063 (49.9) | 448 (49.4) | 0.7922 |
| ASA Class (%) | |  |  | 0.7 |
| 1 | 89 (0.9) | 1056 (5.3) | 5 (0.6) |  |
| 2 | 4274 (42.2) | 9561 (47.7) | 185 (20.6) |  |
| 3 | 5311 (52.4) | 8695 (43.4) | 614 (68.4) |  |
| 4 | 462 (4.6) | 710 (3.5) | 91 (10.1) |  |
| Emergent Case (%) | 1133 (11.1) | 1998 (9.9) | 276 (30.5) | 0 |
| Inpatient (%) | 9068 (88.8) | 18102 (89.8) | 890 (98.1) | 0 |
| Surgical Service (%) | |  |  | 0 |
| General Surgery | 1764 (17.3) | 4975 (24.7) | 239 (26.4) |  |
| Thoracic Surgery | 248 (2.4) | 540 (2.7) | 16 (1.8) |  |
| Vascular Surgery | 723 (7.1) | 1081 (5.4) | 127 (14.0) |  |
| Genito-Urologic Surgery | 1183 (11.6) | 2356 (11.7) | 47 (5.2) |  |
| Transplant Surgery | 239 (2.3) | 1122 (5.6) | 26 (2.9) |  |
| Primary Language (%) | |  |  | 0 |
| English | 239 (2.3) | 18301 (90.7) | 799 (88.1) |  |
| Spanish | 341 (3.3) | 893 (4.4) | 33 (3.6) |  |
| Chinese - Cantonese | 242 (2.4) | 282 (1.4) | 31 (3.4) |  |
| Unable to spell WORLD backwards (%) | 841 (20.5) | 1135 (13.8) | 184 (48.0) | 0 |
| Not oriented to place (%) | 313 (7.6) | 391 (4.7) | 93 (24.3) | 0 |
| History of Diabetes (%) | 2407 (23.6) | 3987 (19.8) | 312 (34.4) | 0 |
| History of Chronic Kidney Disease (%) | 1041 (10.2) | 2143 (10.6) | 135 (14.9) | 0 |
| History of Heart Failure(%) | 608 (6.0) | 922 (4.6) | 91 (10.0) | 0 |
| History of Smoking (%) | 245 (2.4) | 820 (4.1) | 66 (7.3) | 0 |
| *Abbreviations: SD, standard deviation; ASA, American Society of Anesthesiologists* | | | | |

**Supplementary Figure S2.2:** Receiver Operating Characteristic (ROC) Curve for XGBoost, Neural Network, ML hybrid regression, Clinician-guided regression, and AWOL-S models (Neurosurgery Patients Excluded)


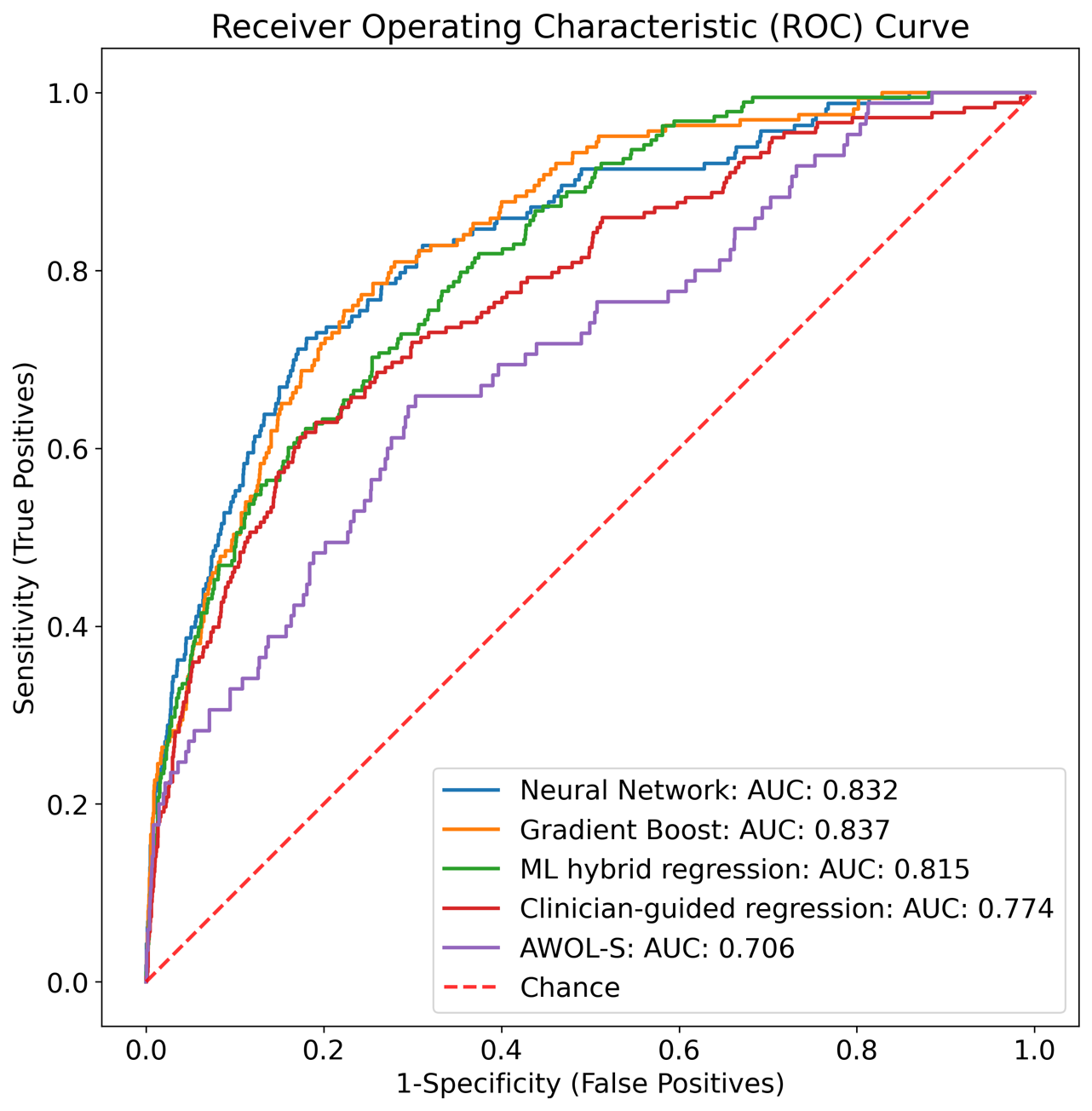


Neural Network: AUC: 0.83

XGBoost: AUC: 0.84

ML hybrid regression: AUC: 0.81

Clinician-guided regression: AUC: 0.75

AWOL-S: AUC: 0.71

Chance

**Supplementary Table 2.2:** Confidence Intervals for AUC-ROC

| ***Model*** | ***AUC-ROC***  ***[95% CI] by cross validation*** | ***AUC-ROC***  ***[95% CI] by DeLong’s method*** |
| --- | --- | --- |
| Neural Network | 0.83 [0.79-0.87] | 0.83 [0.79-0.86] |
| XGBoost | 0.84 [0.81-0.87] | 0.84 [0.81-0.87] |
| ML Hybrid Logistic Regression | 0.80 [0.76-0.83] | 0.81 [0.78-0.84] |
| Expert-Clinician Logistic Regression | 0.76 [0.73-0.80] | 0.75 [0.72-0.79] |
| AWOL-S | n/a | 0.71 [0.65-0.77] |
| *Abbreviations: AUC-ROC, area under the receiver operating characteristics curve; CI, confidence interval; AWOL-S, Age, WORLD backward, Orientation to place, iLlness severity scale, Surgery-specific risk* | | |

**Supplementary Figure S2.3:** Feature Importance Summary of XGBoost Model (Neurosurgery Patients Excluded)


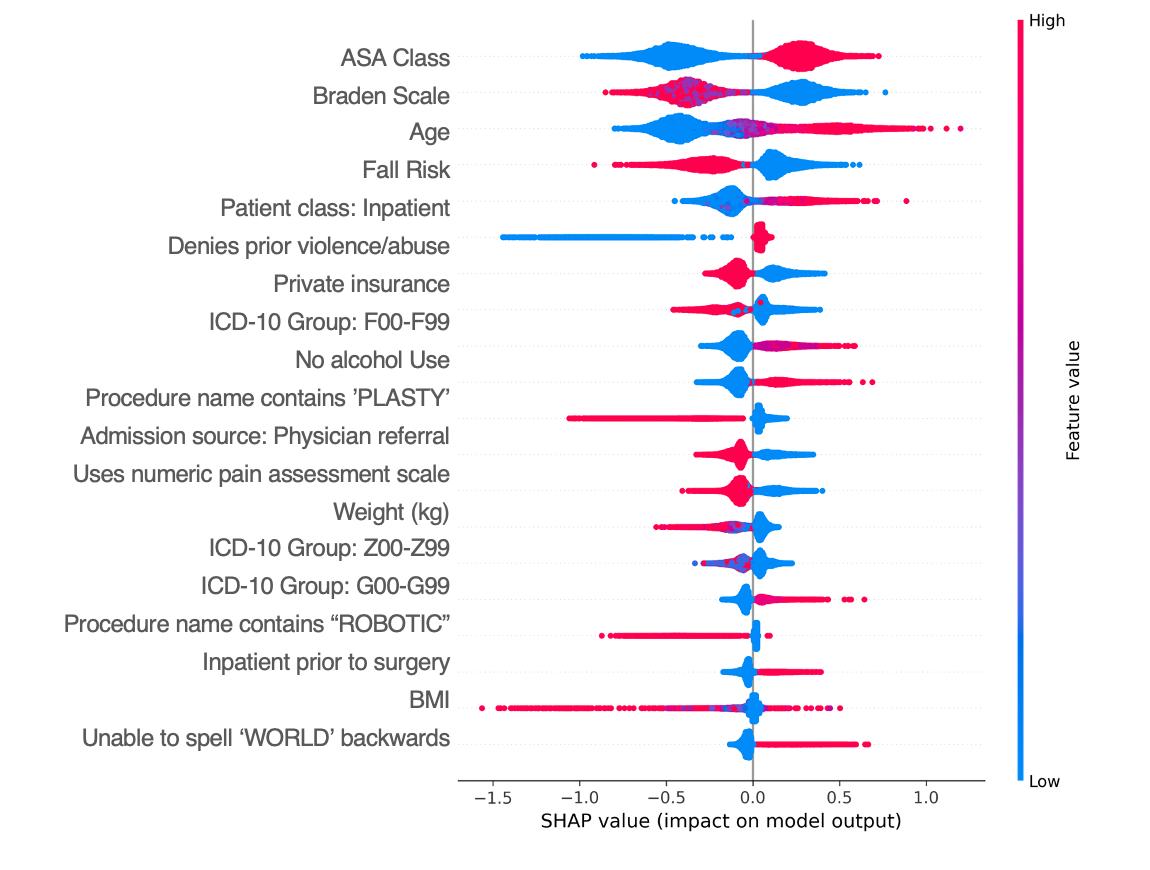


Top 20 most influential variables used by XGBoost (Neurosurgery patients excluded). *Interpretation*: *Each dot represents an individual instance. Red dots indicate higher numeric value or yes/present for categorical variables, and blue dots indicate lower numeric value or no/absent. Dots on the right side of the y-axis indicate a higher impact on the model to predict delirium, and dots to the left of the y-axis indicate features that are protective against prediction of delirium. Dots with the same impact value are plotted on top of each other to create a vertical distribution.*

*Abbreviations: ASA, American Society of Anesthesiologists; ICD-10, International Classification of Diseases, 10th revision; ICD-10 F00-F99, mental and behavioral disorders; preop, preoperative; ICD-10 C00-D48, neoplasms; ICD-10 G00-G99, diseases of the nervous system; BMI, body mass index*

**Supplementary Table S2.3:** Comparison of most important variables chosen by XGBoost (left) and Neural Network (right), Neurosurgery patients excluded. Variables are listed alphabetically. For XGBoost, direction of effect is noted in a separate column. Variables denoted by * are unique to the respective model.

| ***XGBoost*** | | ***Neural Network*** |
| --- | --- | --- |
| *Variable* | *Direction of effect* | *Variable* |
| Ability to answer violence/abuse assessment | Yes = Protective factor | Ability to answer violence/abuse assessment |
| Ability to spell ‘WORLD’ backwards | Yes = Protective factor | Ability to spell ‘WORLD’ backwards* |
| Age | High = Risk factor | Age |
| ASA Class | High = Risk factor | ASA Class |
| Braden Scale | Lower score (Higher pressure ulcer risk) = Risk factor | Braden Scale |
| BMI* | High = Risk factor | Fall risk |
| Fall risk | Yes = Risk factor | General surgery service* |
| History of alcohol use* | Yes = Protective factor | History of substance use* |
| History of mental and behavioral disorders (ICD10 group F00-F99 | Yes = Protective factor | History of mental and behavioral disorders (ICD10 group F00-F99) |
| History of diseases in the nervous system (ICD10 group G00-G99)* | Yes = Risk factor | History of diseases in the cardiovascular system (ICD10 group I00-I99)* |
| History of factors influencing health status (ICD10 group Z00-G99)* | Yes = Risk factor | History of Type 2 Diabetes* |
| Inpatient status | Yes = Risk factor | Inpatient status |
| Name of surgical procedure ending in the phrase ‘-plasty’ | Yes = Protective factor | Name of surgical procedure ending in the phrase ‘-plasty’ |
| Name of surgical procedure containing the phrase ‘robotic’* | Yes = Protective factor | Number of prior anesthetics in past 3 years* |
| Patient Health Insurance Source | Private = Protective factor | Payor |
| Physician referral: admissions source | Yes = Protective factor | Predicted Case Length* |
| Use of numeric scale for preoperative pain assessment | Yes = Protective factor | Preoperative pain score |
| Weight | High = Protective factor | Weight |
| *Abbreviations: ASA, American Society of Anesthesiologists; ERAS, Enhanced Recovery After Surgery; ICD, International Classification of Diseases* | | |

**Supplementary Table S2.4:** Multivariable logistic regression using variables selected by expert clinicians^1^ (Neurosurgery Patients Excluded)

| ***Multivariate Logistic Regression using Expert-Clinician Variables*** | ***Beta*** | ***Odds Ratio***  ***[95% CI]*** | ***p-value*** |
| --- | --- | --- | --- |
| Age | 0.036944 | 1.04 [1.03-1.04] | <0.001 |
| Male | -0.251141 | 0.78 [0.68-0.90] | <0.001 |
| ASA Class > 3 | 0.830702 | 2.29 [1.92-2.74] | <0.001 |
| Emergency Case | 0.788876 | 2.20 [1.89-2.55] | <0.001 |
| High risk surgical service (Cardiac, Thoracic, Vascular) | 0.082873 | 1.09 [0.88-1.32] | 0.414 |
| Primary Language not English | -0.201329 | 0.82 [0.651-1.02] | 0.077 |
| Pre-existing cognitive impairment (unable to spell ‘WORLD’ backwards) | 1.032052 | 2.81 [2.32-3.38] | <0.001 |
| Dependency on ADLs | 0.247502 | 1.28 [0.90-1.79] | 0.156 |
| Visual or hearing impairment | 0.247327 | 1.28 [0.85-1.88] | 0.217 |
| Patient-reported history of falls | 0.264582 | 1.30 [0.90-1.84] | 0.142 |
| Evidence of malnutrition (tube feeds, TPN, pressure ulcers) | 0.403437 | 1.5 [0.92-2.36] | 0.091 |
| History of diabetes (T1DM and T2DM) | 0.298051 | 1.35 [1.15-1.57] | <0.001 |
| History of heart failure (ICD-10 I50) | 0.095348 | 1.10 [0.85-1.40] | 0.445 |
| History of CKD (ICD-10 N18) | -0.174145 | 0.84 [0.68-1.03 | 0.097 |
| History of dyspnea (ICD-10 R06) | -0.287784 | 0.75 [0.427-1.22] | 0.281 |
| History of sepsis (ICD-10 A41 or R78) | 0.479526 | 1.62 [0.97-2.55] | 0.051 |
| Patient reported history of smoking (Yes, Prior, Quit) | 0.315924 | 1.37 [1.18-1.59] | <0.001 |
| *Abbreviations: ASA, American Society of Anesthesiologists; ADL, activities of daily living; TPN, total parenteral nutrition; T1DM, type 1 diabetes mellitus; T2DM, type 2 diabetes mellitus; ICD, International Classification of Diseases; CKD, chronic kidney disease* | | | |

^1^Berian JR, Zhou L, Russell MM, et al. Postoperative Delirium as a Target for Surgical Quality Improvement. *Ann Surg*. 2018;268(1):93-99. doi:10.1097/SLA.0000000000002436.

**Supplementary Table S2.5**: Multivariable logistic regression using variables chosen by the XGBoost algorithm (Neurosurgery Patients Excluded)

| ***Multivariate Logistic Regression using Machine-Learning Derived Variables*** | ***Beta*** | ***Odds Ratio***  ***[95% CI]*** | ***p-value*** |
| --- | --- | --- | --- |
| Age | 0.030 | 1.03 [1.02-1.03] | <0.001 |
| ASA Class | 0.623 | 1.86 [1.56-2.23] | <0.001 |
| BMI | -0.0041 | 1.00 [0.979-1.00] | 0.591 |
| Braden Scale | -0.195 | 0.82 [0.80-0.84] | <0.001 |
| Ability to spell ‘WORLD’ backwards | 0.647 | 1.91 [1.56-2.33] | <0.001 |
| Fall risk | -0.0831 | 0.920 [0.26-1.32] | 0.661 |
| History of alcohol use | 0.017 | 1.02 [0.86-1.20] | 0.837 |
| History of mental and behavioral disorders (ICD10 group F00-F99) | 0.281 | 1.32 [1.22-1.43] | <0.001 |
| History of diseases in the nervous system (ICD10 group G00-G99) | 0.0896 | 1.09 [1.00-1.19] | 0.042 |
| History of factors influencing health status (ICD10 group Z00-Z99) | -0.126 | 0.88 [0.82-0.94] | <0.01 |
| Inpatient status (Patient class and Prior location) | 1.65 | 5.21 [3.21-9.17] | <0.001 |
| Name of surgical procedure ending in the phrase ‘-PLASTY’ | -0.979 | 0.38 [0.28-0.49] | <0.001 |
| Name of surgical procedure containing the phrase ‘ROBOTIC’ | -1.06 | 0.35 [0.20-0.55] | <0.001 |
| Payor | -0.395 | 0.68 [0.56-0.81] | <0.001 |
| Preoperative violence or abuse assessment | -0.189 | 0.83 [0.68-1.02] | 0.072 |
| Use of numeric scale for preoperative pain assessment | -0.601 | 0.59 [0.47-0.63] | <0.001 |
| Weight | -0.0085 | 0.99 [0.992-0.996] | <0.01 |
| Age | 0.030 | 1.03 [1.02-1.03] | <0.001 |
| *Abbreviations: ASA, American Society of Anesthesiologists; BMI, body mass index; ICD, International Classification of Dieases* | | | |
